# The burden of thyroid cancer and its association with national development levels in Asia: evidence from 47 countries (1990–2021)

**DOI:** 10.3389/fonc.2025.1632221

**Published:** 2025-08-13

**Authors:** Wenyi Qin, Jiong Lin, Zhi Zhang, Tuo Xu, Siyi Li, Weijie Chen, Muge Liu, Baibei Li, Haiqing Luo, Yu Lili

**Affiliations:** ^1^ Faculty of Chinese Medicine, Macau University of Science and Technology, Macau, Macau SAR, China; ^2^ Department of Vascular and Thyroid Surgery, Affiliated Hospital of Guangdong Medical University, Zhanjiang, China; ^3^ State Key Laboratory of Quality Research in Chinese Medicines, Macau University of Science and Technology, Macau, Macau SAR, China

**Keywords:** thyroid cancer, incidence, mortality, mortality-to-incidence ratio, human development index

## Abstract

**Objective:**

This study examines the trends in thyroid cancer incidence and mortality and explores their relationship with national development levels.

**Methods:**

Data on thyroid cancer from 47 countries were sourced from the Global Burden of Disease 2021 study, covering the years 1990–2021. National development was measured using the Human Development Index (HDI), Social Progress Imperative (SPI), Nutrition and Medical Care (NMC), the Density of Doctors per 100,000 population (DOD), and Personal Healthcare Spending (PHS). Correlations between these indicators and thyroid cancer burden were analyzed using scatterplot matrices, heat maps, and principal component analysis (PCA). The mortality-to-incidence ratio (MIR) served as a proxy for thyroid cancer survival rates.

**Results:**

In 2021, thyroid cancer caused 44,798 deaths globally, up from 21,893 in 1990. Asia accounted for 62.8% of the cases. Among the working-age population, deaths ranged between 10,477 and 27,187, while incidence rates rose from 1.41 per 100,000 in 1990 to 3.36 per 100,000 in 2021. Nine out of the top ten countries in terms of incidence were classified as high or very high in HDI. The incidence of thyroid cancer correlated positively with HDI (Corr = 0.365), SPI (Corr = 0.384), and NMC (Corr = 0.332), particularly among men. Mortality, however, showed a negative correlation with HDI (Corr = -0.401), NMC (Corr = -0.437), and PHS (Corr = -0.446). The global MIR dropped from 0.135 in 1990 to 0.068 in 2021, though Asia saw a 21.1% rise in mortality and a 138% increase in incidence among the working-age population.

**Conclusion:**

The rising incidence of thyroid cancer in Asia, particularly in developed nations, has contributed to a global increase, though improved prognoses have led to better survival outcomes over time.

## Introduction

Thyroid cancer (TC) is one of the most common cancers worldwide and is prevalent among young people. The incidence of TC increased from 4.8 to 14.9 cases per 100,000 people, then stabilized and appeared to decline again to about 13.5 cases per 100,000 in 2018 (1). Due to its high incidence in both developed and developing countries, TC is estimated to cause 500,000 to 600,000 new cases and more than 40,000 deaths globally each year (2). The main histological types of TC are papillary thyroid carcinoma (PTC), follicular thyroid carcinoma (FTC), Hürthle cell thyroid carcinoma (HCTC), medullary thyroid carcinoma (MTC), and anaplastic thyroid carcinoma (ATC), accounting for 80.2%, 11.4%, 3.1%, 3.5%, and 1.7% of all TCs, respectively (3). Studying the epidemiology of TC can help identify priority screening populations, ultimately improving public health outcomes.

TC is one of the most common tumors among young and middle-aged populations, with a higher prevalence in females (4, 5). Some studies have found that the rapid increase in TC incidence is primarily seen in PTC (6). In many Asian countries, the incidence of female PTC rose sharply after 2000; however, in many American and European countries, the incidence has stabilized since around 2009 (6). Therefore, studying TC incidence and mortality in Asia over the past three decades would better reflect the region’s epidemiological trends for this disease.

This paper examines the burden (incidence and mortality) of TC in 47 Asian countries over the period 1990–2021, with a focus on the temporal patterns of TC burden and their association with national development levels. Estimates of the TC burden were obtained from the Global Burden of Disease (GBD) 2021 study (7). National development and medical capacity were measured using the Human Development Index (HDI), the Inequality-adjusted Human Development Index (IHDI), the Social Progress Imperative (SPI), Nutrition and Medical Care (NMC), the Density of Doctors per 100,000 population (DOD), and Personal Healthcare Spending (PHS). HDI and IHDI data were compiled by the United Nations Development Program (UNDP), while SPI and NMC data were sourced from the Social Progress Imperative website, which provides official reference data for the GBD database. DOD and PHS data were obtained from official World Bank and World Health Organization (WHO) sources. We investigate the burden of TC across a large number of countries (47 countries) and analyze temporal patterns in TC incidence and mortality. In addition to examining these factors, we also investigate the mortality-to-incidence ratio (MIR) of TC, which has been shown to be a surrogate for 5-year survival (8). In this study, we use multidimensional development indicators, including the HDI and IHDI, as a comparison and complement to the SDI used by the GBD. While some researchers consider the SDI a composite measure of income, education, and fertility, the HDI is based on income, education, and life expectancy. Since the cancer burden is positively correlated with age, life expectancy appears to be a more relevant indicator than fertility when describing cancer burden, hence the inclusion of the HDI (9). Examining past trends in morbidity, mortality, and survival (expressed as the MIR) in relation to the HDI provides insight into the progress we are making in combating a disease that contributes significantly to global mortality and morbidity.

TC has been shown to be increasingly prevalent among young people in developed countries (1),and recent studies confirm that its global profile is characterized by worse prognoses in males and in low SDI areas, with females experiencing a higher incidence compared to males (10). However, there is a lack of studies on the incidence and mortality characteristics of TC in the key working-age population (20–54 years), which is increasingly likely to undergo routine medical check-ups and benefit from higher incomes as working conditions improve (11). These factors may increase the probability of detecting various diseases, including TC, and potentially improve disease prognosis (12, 13). Asia, the world’s most populous continent, encompasses a wide range of economic conditions—from highly developed countries to war-torn regions—providing a broad social spectrum for studying the relationship between TC and development.

## Methods

The estimates of TC incidence and mortality (for ages 20–54, both male and female) were obtained from the Global Burden of Disease (GBD) 2021 study for the period 1990–2021 (7), available at https://vizhub.healthdata.org/gbd-results/. The GBD 2021 study analyzed disease and injury burdens by estimating incidence, prevalence, mortality, years lived with disability (YLDs), disability-adjusted life years (DALYs), and healthy life expectancy (HALE) for 371 diseases and injuries, using 100,983 data sources from 195 countries during the 1990–2021 period. These data sources included a variety of platforms, such as autopsies, vital registration systems, household surveys, censuses, disease-specific registries, and health service contact data. The GBD procured data from various databases and applied multiple modeling steps to produce epidemiological estimates (14, 15). We will briefly discuss how the GBD produces estimates of TC incidence and mortality. First, GBD methodology processes incidence and mortality data from cancer registries, then matches the processed data by cancer type, age, sex, year, and location to generate crude MIRs. These crude MIRs are then used as inputs in a three-step modeling approach that employs general spatio-temporal Gaussian process regression (ST-GPR). Socio-demographic indices (SDIs) are used as covariates in a linear mixed-effects model with a log-link function to obtain the final MIRs. In the third step, the incidence rates from cancer registries are multiplied by the MIRs derived in the second step to estimate mortality. These mortality estimates are then input into the cause-of-death pooled model (CodeM) to generate final mortality estimates (15, 16). The CodeM model is designed to derive cause-specific mortality estimates using an ensemble modeling approach, where the predictive validity of numerous models is tested before deriving the final estimates. Only covariates that are reasonably related to a particular cause of death are selected for this model. Some covariates used by CodeM for TC mortality modeling include education, lagged distribution of income, socio-demographic indices, and indices of healthcare accessibility and quality. The final morbidity estimate is obtained by dividing the final mortality estimate by the MIRs derived in the second step (16).

In this study, we obtained estimates of TC incidence and mortality for the period 1990 to 2021 and for 2021, as well as for 2021 specifically. We used incidence and mortality rates for the 20–54 age group to describe the burden of TC in the primary working population. TC survival was expressed as the MIR, which has been shown to be a reasonable surrogate for survival across various tumors (8, 17). Although the MIR is not a perfect representation of survival, it provides valuable insights into relative survival rates of TC across different countries. The MIR was calculated directly from the crude mortality-to-incidence ratio provided by the GBD 2021 report. In this study, we examine TC in Asian countries, including the Democratic People’s Republic of Korea (DPRK). Consistent with GBD methodology, we report morbidity and mortality estimates with 95% uncertainty intervals.

A country’s level of development is measured by its HDI—a composite of three indicators: health (life expectancy at birth), education (average and expected years of schooling), and income (gross national income per capita). Each indicator is scaled from 0 to 1, with 0 representing the lowest and 1 the highest value. The final HDI is calculated as the geometric mean of the health, education, and income indices. The IHDI, based on the distributionally sensitive composite indices proposed by Foster, Lopez-Calva, and Szekely (18), adjusts the HDI for inequality. It is computed as a geometric average of inequality-adjusted dimensional indices. The IHDI “discounts” the mean of each dimension based on the level of inequality, so the IHDI equals the HDI when there is no inequality, but falls below the HDI as inequality increases. In this sense, the IHDI measures human development after accounting for inequality. HDI and IHDI data are obtained from the United Nations Development Program (UNDP) database. For descriptive and analytical purposes, countries are classified into four categories based on the UNDP classification: very high (HDI > 0.800, 17 countries), high (0.700 < HDI < 0.799, 16 countries), medium (0.550 < HDI < 0.669, 10 countries), and low (HDI < 0.550, 3 countries). HDI data is unavailable for the DPRK, so its level of development will be analyzed using other indicators.

The SPI measures the extent to which countries meet the social and environmental needs of their citizens. Fifty-four indicators across three dimensions—basic human needs, foundations of well-being, and opportunities for progress—highlight the relative performance of countries. The index is published by the non-profit organization Social Progress Imperative. Social and environmental factors include health (such as housing and sanitation), equality, inclusion, sustainability, and personal freedom and security. The NMC component, part of the SPI, includes scores for child mortality, child stunting, diets low in fruits and vegetables, infectious diseases, maternal mortality, and undernourishment. Data for the SPI and NMC were accessed from the Social Progress Imperative website.

The DOD and PHS reflect a country’s basic health status and healthcare expenditure, providing a more intuitive picture of healthcare. We use these indicators as controls and complements to the aforementioned statistical indicators. DOD and PHS data were obtained from the WHO and World Bank databases. All data analyses were conducted using Microsoft Excel and R Studio 4.2.

Employing the methodology described, three primary outcome variables(incidence, mortality, and MIR) are quantified and disaggregated by development indices and sex.

## Results

**Figure 1:**
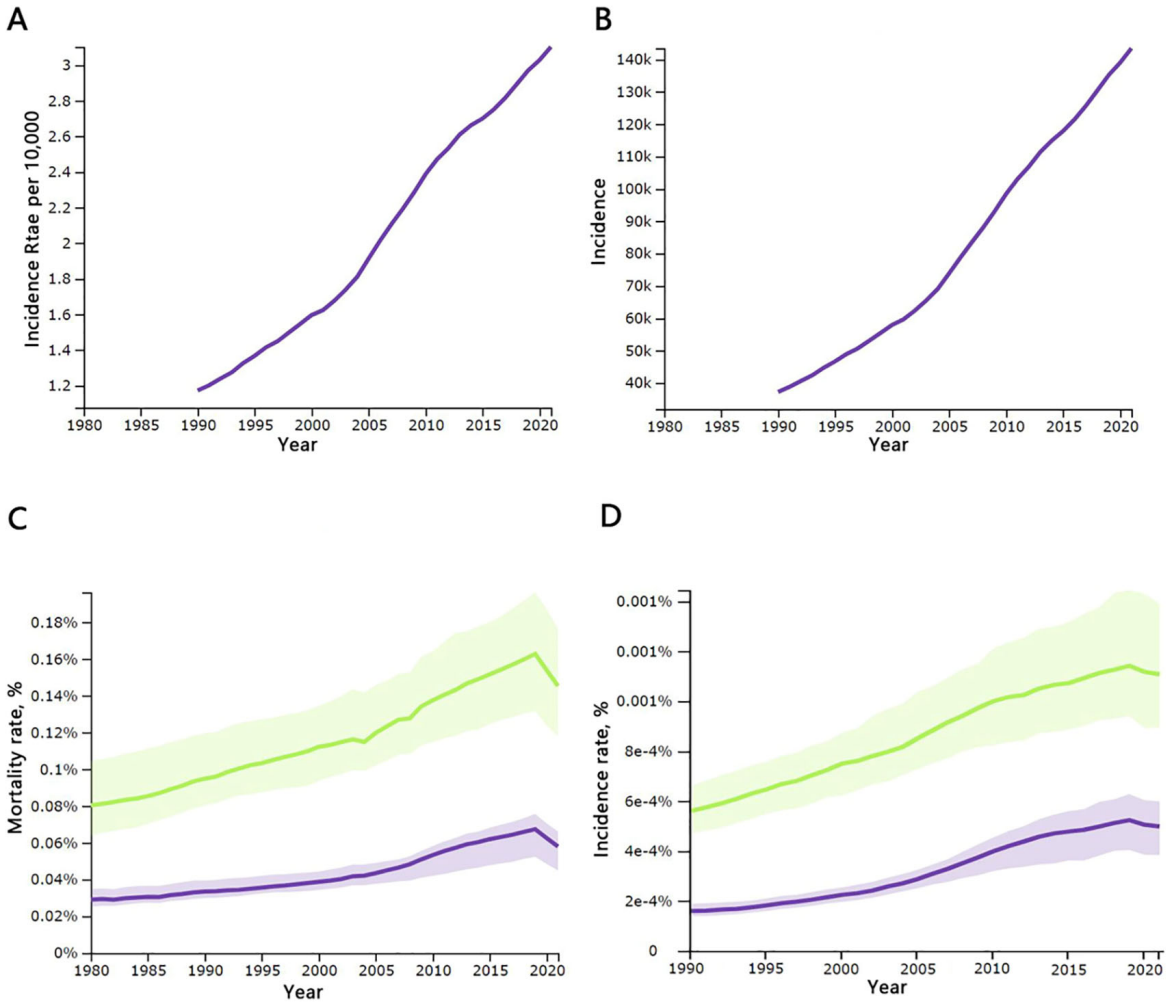
Temporal changes in key global thyroid cancer indicators, 1990-2021: **(A)** incidence rates per 10,000 population of all ages; **(B)** incidence rates of all ages; **(C)** mortality rates for males and females aged 20–54 years; **(D)** morbidity rates for males and females aged 20–54 years. Incidence, number of cases; mortality, number of deaths; males are shown with a green line, females with a purple line; source: Global Burden of Disease 2021 study.

In total, thyroid cancer (TC) resulted in 44,798 [95% uncertainty interval (UI), 39,924–48,541] deaths in 2021, up from 21,893 [20,437–24,108] in 1990 across all age groups. Asia accounted for 62.8 percent of the world’s thyroid cancer cases in 2021. Among the working-age population, this number varied between 10,477[9,394–12,252] and 27,187[23,128–30,091], and the incidence of thyroid cancer has changed from 1.41 per 100,000 [1.23–1.63] in 1990 to 3.36 per 100,000 [2.81–3.90] in 2021. The mortality and incidence rates were significantly higher in females than in males, as shown in **Table 1** and **Figure 1**.

**Table 1:** Country-wise thyroid cancer burden in the 20–54 age group for 2021.

Location	HDI	IHDI	SPI	NMC	DOD	PHS	Mortality	Mortality (Male)	Mortality (Female)	Incidence	Incidence (Male)	Incidence (Female)	MIR
Afghanistan	0.46	0.30	32.15	46.27	2.54	81.32	0.30	0.10	0.51	3.07	0.56	5.67	0.10
Armenia	0.79	0.72	73.21	92.49	31.17	613.00	0.26	0.27	0.26	3.52	2.19	4.77	0.07
Azerbaijan	0.76	0.71	62.67	89.42	30.93	249.00	0.13	0.12	0.13	1.26	0.76	1.76	0.10
Bahrain	0.89	NA	66.10	94.67	8.42	1146.47	0.13	0.08	0.23	5.08	2.08	11.40	0.03
Bangladesh	0.67	0.47	54.60	67.69	6.70	57.94	0.23	0.18	0.28	2.31	1.10	3.42	0.10
Bhutan	0.68	0.47	65.49	73.28	5.52	120.43	0.24	0.19	0.29	2.14	1.14	3.28	0.11
Brunei	0.82	0.73	NA	82.19	19.13	693.41	0.20	0.13	0.29	3.69	1.14	6.66	0.06
Cambodia	0.60	0.44	55.36	68.23	2.14	63.79	0.35	0.19	0.50	3.32	1.04	5.54	0.10
China	0.79	0.66	67.61	92.34	25.18	670.51	0.17	0.21	0.12	3.39	2.86	3.95	0.05
Cyprus	0.91	0.83	81.88	91.28	35.52	2990.00	0.11	0.14	0.08	2.98	3.07	2.89	0.04
Georgia	0.81	0.73	72.62	87.56	56.13	484.00	0.41	0.33	0.50	4.75	2.19	7.32	0.09
India	0.64	0.44	58.06	66.97	7.27	74.00	0.26	0.19	0.33	2.39	1.15	3.69	0.11
Indonesia	0.71	0.59	67.22	73.86	6.90	160.64	0.28	0.19	0.36	2.70	1.12	4.32	0.10
Iran	0.78	0.58	60.30	92.36	15.14	392.54	0.11	0.10	0.11	4.51	2.85	6.22	0.02
Iraq	0.67	0.52	57.76	80.64	10.12	248.92	0.20	0.17	0.23	5.31	2.98	7.91	0.04
Israel	0.92	0.81	81.68	96.74	37.13	4339.00	0.14	0.17	0.11	3.31	3.09	3.53	0.04
Japan	0.92	0.84	85.52	90.67	26.14	4347.00	0.11	0.09	0.12	5.42	2.13	8.82	0.02
Jordan	0.74	0.62	66.27	88.09	25.13	299.07	0.14	0.13	0.16	4.81	2.96	7.09	0.03
Kazakhstan	0.80	0.73	69.73	91.11	40.28	403.00	0.27	0.21	0.31	3.60	1.58	5.54	0.07
Kuwait	0.85	NA	73.44	93.95	22.93	1860.78	0.13	0.12	0.13	6.55	4.33	9.02	0.02
Kyrgyzstan	0.70	0.63	66.20	86.25	21.45	73.00	0.22	0.13	0.30	2.63	0.86	4.37	0.08
Laos	0.62	0.47	53.05	71.09	3.27	68.88	0.29	0.17	0.41	2.36	0.81	3.93	0.12
Lebanon	0.72	NA	64.30	93.59	26.17	307.13	0.13	0.12	0.13	5.46	3.45	7.47	0.02
Malaysia	0.81	0.69	73.83	85.14	23.16	487.01	0.25	0.20	0.31	4.42	2.06	7.04	0.06
Maldives	0.76	0.60	67.75	84.17	21.61	1039.00	0.06	0.06	0.07	1.14	0.74	1.93	0.06
Mongolia	0.74	0.65	66.15	79.45	38.74	315.58	0.25	0.22	0.28	2.07	1.09	3.03	0.12
Myanmar	0.61	0.48	51.19	72.75	7.51	65.00	0.28	0.16	0.39	2.62	0.84	4.26	0.11
Nepal	0.60	0.42	58.10	74.14	8.67	65.00	0.27	0.20	0.32	2.27	1.02	3.28	0.12
North Korea	NA	NA	NA	68.69	36.71	NA	0.23	0.19	0.27	3.27	1.56	5.14	0.07
Oman	0.82	0.72	68.52	90.35	20.58	852.62	0.07	0.06	0.08	2.62	1.70	4.53	0.03
Pakistan	0.54	0.36	48.87	58.63	10.84	43.09	0.48	0.26	0.70	3.53	1.16	5.91	0.14
Palestine	0.72	0.59	NA	NA	21.68	NA	0.12	0.09	0.16	3.42	1.57	5.34	0.04
Philippines	0.71	0.59	66.16	76.72	7.86	178.00	0.41	0.28	0.54	4.20	1.69	6.82	0.10
Qatar	0.88	NA	69.29	95.17	24.99	1934.08	0.10	0.10	0.10	4.66	3.83	7.29	0.02
Saudi Arabia	0.88	NA	65.58	89.40	30.77	1442.00	0.29	0.22	0.39	9.87	4.83	17.54	0.03
Singapore	0.95	0.83	84.21	94.58	25.96	3969.89	0.07	0.06	0.08	3.06	1.26	4.96	0.02
South Korea	0.93	0.84	85.26	94.57	25.17	3123.95	0.13	0.12	0.15	6.51	2.61	10.73	0.02
Sri Lanka	0.78	0.63	66.67	83.33	11.92	166.00	0.19	0.16	0.21	3.86	1.91	5.69	0.05
Syria	0.56	NA	48.14	75.00	11.86	63.14	0.14	0.12	0.15	3.91	2.06	5.40	0.03
Tajikistan	0.68	0.59	57.45	79.09	21.33	73.00	0.00	0.00	0.00	0.01	0.00	0.02	0.15
Thailand	0.80	0.68	70.67	85.50	9.28	364.37	0.27	0.25	0.29	5.67	3.18	8.03	0.05
Turkey	0.86	0.72	66.23	94.67	21.65	441.00	0.15	0.15	0.15	4.93	3.36	6.54	0.03
Turkmenistan	0.74	NA	60.47	86.95	21.44	565.26	0.22	0.20	0.24	1.93	1.08	2.89	0.11
United Arab Emirates	0.94	0.86	72.92	90.87	29.12	2351.81	0.16	0.13	0.24	3.86	2.39	8.69	0.04
Uzbekistan	0.73	NA	66.88	88.65	28.05	157.00	0.08	0.06	0.10	0.77	0.33	1.21	0.10
Vietnam	0.73	0.61	69.09	82.54	8.33	172.55	0.43	0.32	0.54	8.24	3.45	13.17	0.05
Yemen	0.42	0.29	39.88	54.66	2.94	63.30	0.11	0.06	0.15	1.53	0.56	2.49	0.07

HDI, Human Development Index; IHDI, Inequality-adjusted Human Development Index; SPI, Social Progress Imperative; NMC, Nutrition and Medical Care; DOD, Density of Doctors per 100,000 population; PHS, Personal Healthcare Spending; Incidence, Incidence per 100,000; mortality, Deaths per 100,000; MIR, mortality-to-incidence ratio. Data Source: Global Burden of Disease 2021 study.

### Incidence and mortality of thyroid cancer in 2021

**Figure 2:**
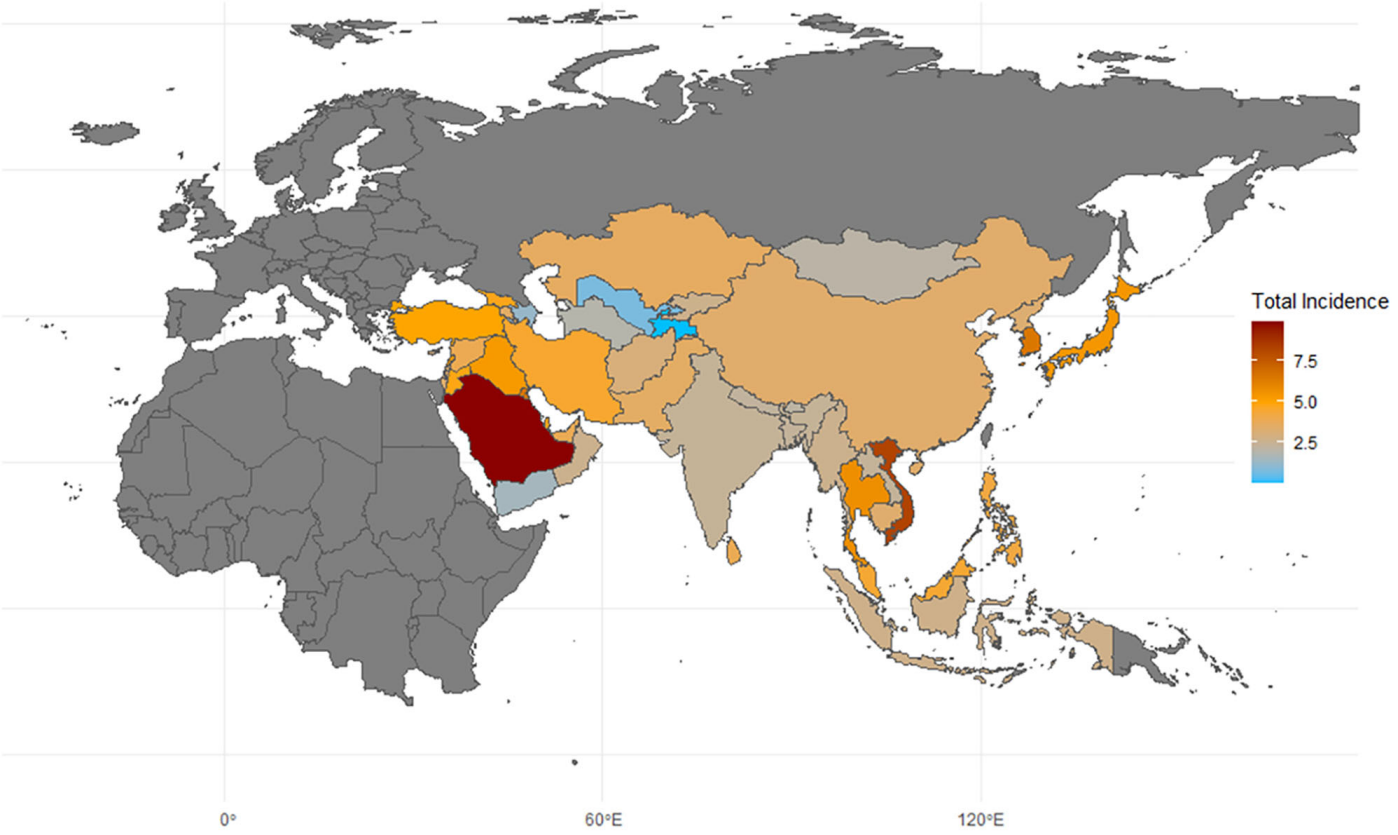
Map of thyroid cancer incidence in asia. 2021; Incidence, incidence per 100,000; source: Global Burden of Disease 2021 study.

In terms of incidence, countries with very high and high HDI levels make up nine of the top ten. Saudi Arabia has the highest incidence rate, at 9.867 per 100,000, which is 303% of the world average of 3.26 per 100,000, followed by Vietnam, with a rate of 8.243 per 100,000, as shown in **Table 1** and **Figure 2**. The analysis of total, male, and female incidence rates in relation to development levels is presented using three methods: a scatterplot matrix, a correlation heat map, and a principal component analysis (PCA) plot. Arrow length of PCA plot reflects variable contribution to principal components (PC1/PC2); angles between arrows indicate correlation strength (acute = positive, obtuse = negative).

The scatterplot matrix displays the relationship between multiple variables by creating a matrix where each cell contains a scatter plot between two variables. This allows us to observe linear or non-linear relationships, correlations, trends, and more. Correlation coefficient values are added to the matrix: coefficients close to 1 or -1 indicate a strong correlation, while values near 0 suggest little to no correlation. Statistical significance is denoted by an asterisk in the upper right corner of the coefficient.

The heat map visually represents the correlation between different variables using color, with the shade indicating the strength of the correlation. This makes it easy to identify which variables have stronger or weaker linear relationships with one another.

A principal component analysis (PCA) plot reduces multidimensional data into a few principal components, helping to reveal underlying structures or patterns in complex, high-dimensional data. The primary goal of a PCA plot is to compress as much raw information (variability in the data) as possible into a few principal components, usually the first two. PCA biplots project both sample points (individuals) and variables onto the same plot, making it easier to visualize the distribution of individuals and the relationships between variables. In the PCA plot, each arrow represents the projection of an original variable into the principal component space. Longer arrows indicate a greater contribution of the variable to the principal components (strong explanatory power), while shorter arrows represent weaker explanatory power. An arrow angle close to 0 degrees suggests a positive correlation between two variables, around 90 degrees indicates no significant correlation, and close to 180 degrees suggests a negative correlation.

**Figure 3:**
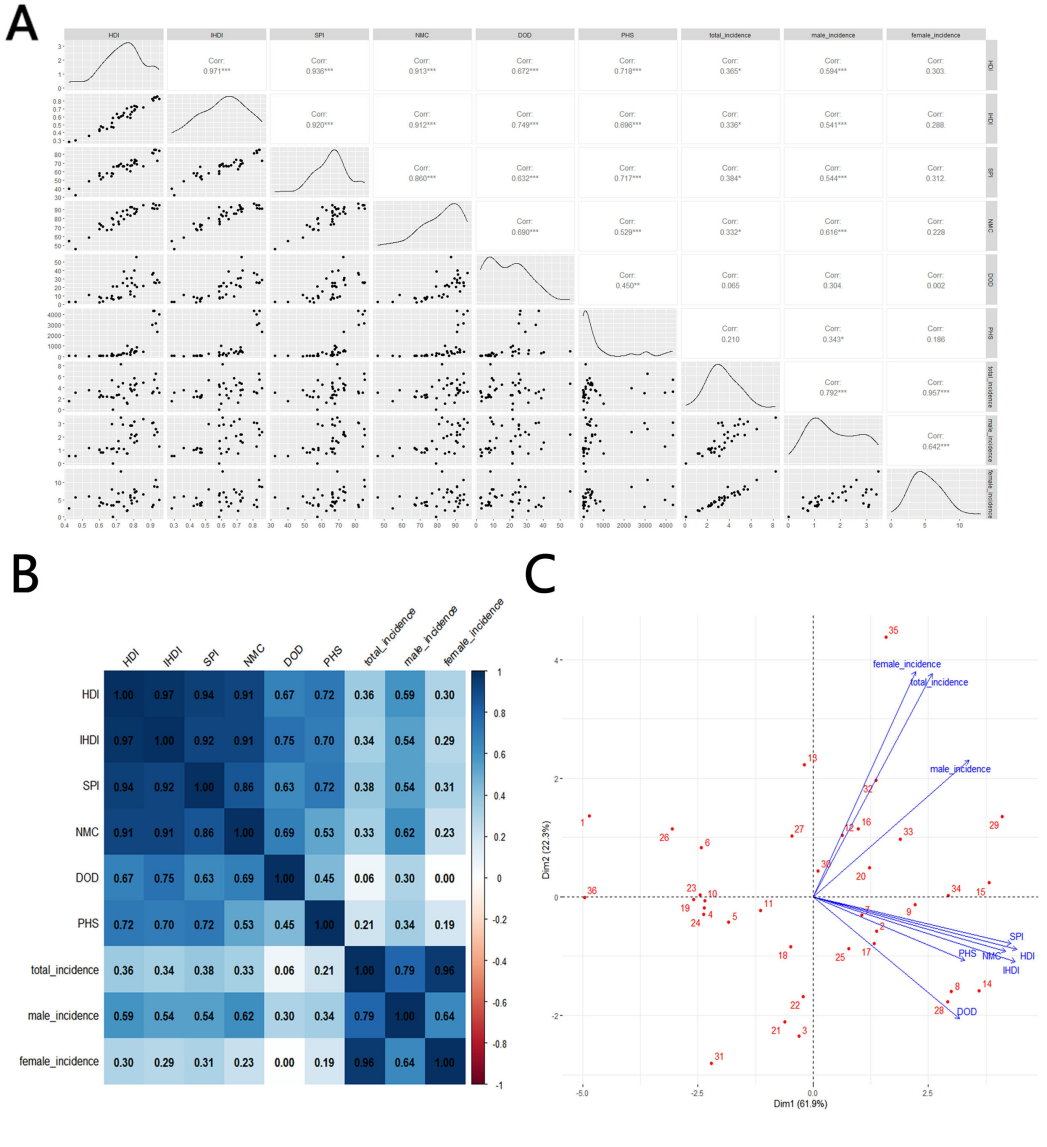
Correlation between thyroid cancer incidence and social development parameters, 2021: **(A)** scatterplot matrix; **(B)**, a correlation heat map; **(C)** principal component analysis plot; HDI, Human Development Index; IHDI, Inequality-adjusted Human Development Index; SPI, Social Progress Imperative; NMC, Nutrition and Medical Care; DOD, Density of Doctors per 100,000 population; PHS, Personal Healthcare Spending; Incidence, Incidence per 100,000; source: Global Burden of Disease 2021 study. An asterisk (*) indicates statistical significance at approximately P = 0.01, two asterisks denote high significance at approximately P = 0.001, and three asterisks signify extreme significance at approximately P = 0.0001.

According to the results, the total incidence rate of TC in the working-age population shows a positive correlation with four indicators: HDI(Corr=0.365^*^), IHDI(Corr=0.336^*^), SPI(Corr=0.384^*^), and NMC(Corr=0.332^*^). Notably, the incidence rate among males is more strongly correlated with these social development indicators, with correlations of 0.594^***^, 0.541^***^, 0.544^***^ and 0.616^***^, respectively. However, there is no significant correlation between the incidence rate among females and these indicators (Corr = 0.303, 0.288, 0.312, and 0.228). The most significant indicator of incidence is SPI(Corr=0.384^*^), while DOD(Corr=0.065), which reflects basic healthcare availability, does not appear to correlate with incidence. The contribution to the total incidence rate comes more from women, which aligns with the general understanding that TC is more common in women than in men, as shown in **Figure 3**.

**Figure 4:**
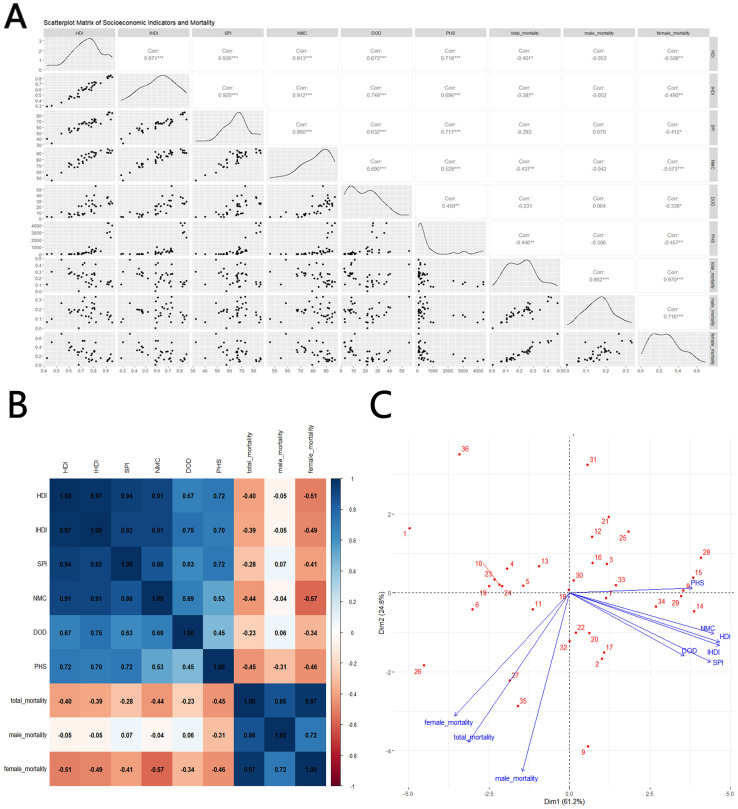
Correlation between thyroid cancer mortality and social development parameters, 2021: **(A)** scatterplot matrix; **(B)** a correlation heat map; **(C)** principal component analysis plot; HDI, Human Development Index; IHDI, Inequality-adjusted Human Development Index; SPI, Social Progress Imperative; NMC, Nutrition and Medical Care; DOD, Density of Doctors per 100,000 population; PHS, Personal Healthcare Spending; Mortality, Mortality per 100,000; source: Global Burden of Disease 2021 study. An asterisk (*) indicates statistical significance at approximately P = 0.01, two asterisks denote high significance at approximately P = 0.001, and three asterisks signify extreme significance at approximately P = 0.0001.

In terms of mortality, HDI(Corr=-0.401^*^), IHDI(Corr=-0.387^*^), NMC(Corr=-0.437^**^) and PHS(Corr=-0.446^**^) shows negative correlations with total mortality, with stronger correlations for NMC and PHS. However, in contrast to the morbidity results, male mortality does not show a meaningful correlation when compares with sociological indicators(Corr=-0.053, -0.052, -0.042 and -0.306), while on the contrary, female mortality is significantly negatively correlated with all indicators, especially NMC(Corr=-0.508^**^, -0.49^**^0, -0.573^***^ and -0.457^**^), as shown in **Figure 4**.

### Mortality, incidence and MIR change of thyroid cancer

**Figure 5:**
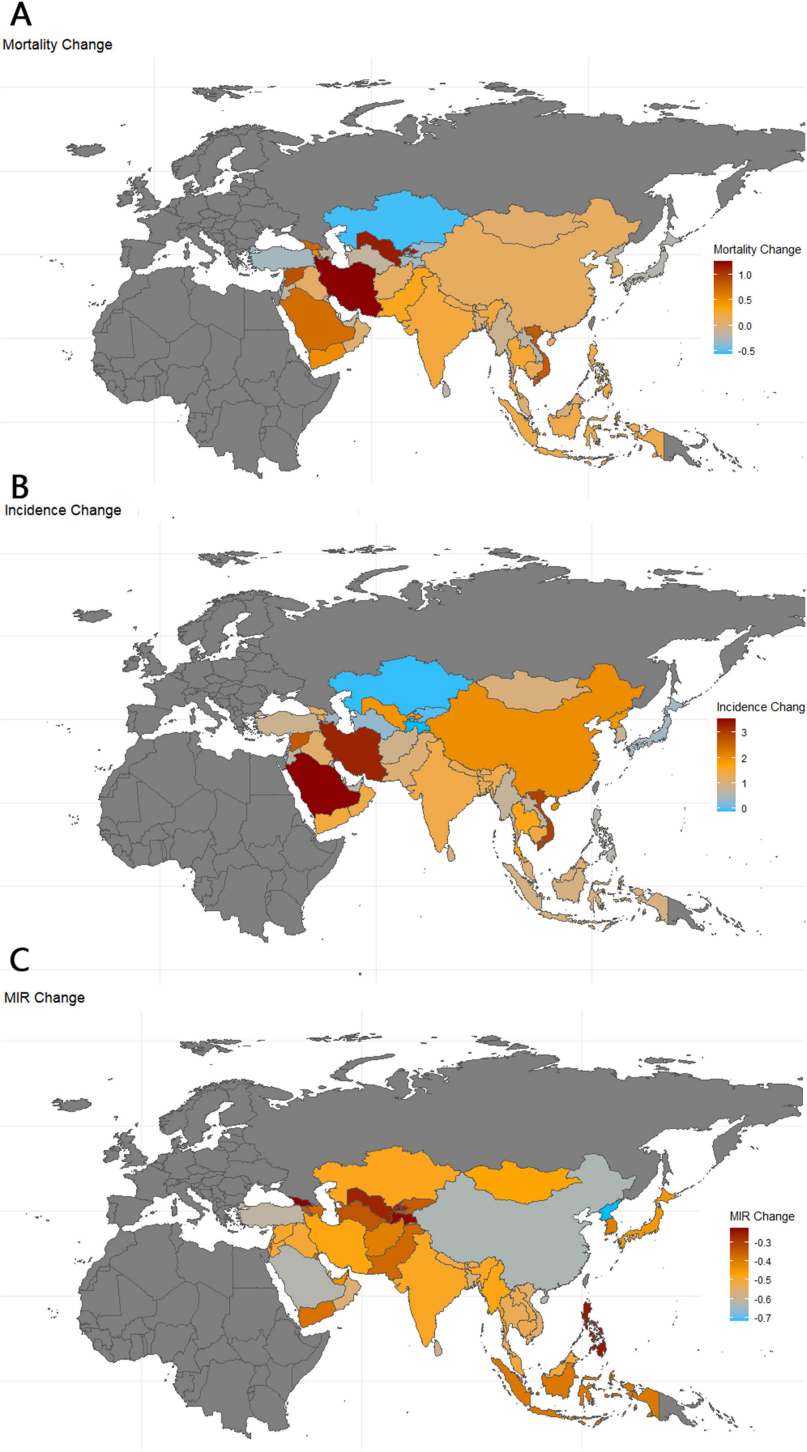
Map of thyroid cancer mortality, incidence and MIR change in Asia. 1990-2021; Incidence, incidence per 100,000; Mortality, Mortality per 100,000;source; MIR, mortality to incidence ratio: Global Burden of Disease 2021 study.

The global MIR declined from 0.135 in 1990 to 0.068 in 2021. According to the data, Asia’s working-age population experienced a 21.1% increase in TC mortality and a 138% increase in incidence. The incidence of TC increased in all countries except Kazakhstan and Tajikistan, where it decreased by 7.1% and 11.7%, respectively. Incidence rates increased in 23 of the 47 countries—4 of these 23 were less-developed countries (low/medium HDI), while 18 were developed countries (high/very high HDI); the DPRK does not have a referable HDI. Mortality rates increased in 24 of the 47 countries—9 of these 24 were less-developed countries (low/medium HDI), while 15 were developed countries (high/very high HDI). MIR declined in all countries, with the largest decreases seen in DPRK (-71.6%), Turkey (-66.2%), Maldives (-63.0%), China (-62.4%), and Saudi Arabia (-60.9%), as shown in **Table 2** and **Figure 5**.

**Table 2:** Country-wise thyroid cancer burden of 20–54 ages from 1990 to 2021.

Location	Development	Mortality change	Mortality change (Male)	Mortality change (Female)	Incidence change	Incidence change (Male)	Incidence change (Female)	HDI change	MIR change
Afghanistan	4.00	0.11	0.24	0.21	0.86	1.04	1.09	0.63	-0.41
Armenia	2.00	0.50	1.02	0.20	1.29	2.42	1.00	0.20	-0.34
Azerbaijan	2.00	-0.14	-0.01	-0.23	0.37	0.66	0.31	NA	-0.37
Bahrain	1.00	-0.03	0.68	-0.18	1.16	2.76	1.09	0.21	-0.55
Bangladesh	3.00	0.00	0.21	-0.13	1.28	1.13	1.64	0.68	-0.56
Bhutan	3.00	0.08	0.38	-0.09	1.27	1.82	1.08	NA	-0.53
Brunei	1.00	0.01	0.46	0.09	0.74	1.25	0.65	0.06	-0.42
Cambodia	3.00	0.16	0.38	0.17	1.41	1.49	1.58	0.58	-0.52
China	2.00	0.11	0.84	-0.35	2.01	4.60	1.21	0.64	-0.63
Cyprus	1.00	-0.49	-0.17	-0.70	0.23	1.10	-0.15	0.24	-0.59
Georgia	1.00	0.73	0.53	0.92	1.23	0.91	1.41	NA	-0.22
India	3.00	0.24	0.47	0.12	1.41	1.78	1.28	0.48	-0.49
Indonesia	2.00	0.19	0.65	0.05	0.96	1.52	0.90	0.36	-0.39
Iran	2.00	1.27	2.09	0.79	3.30	5.41	2.74	0.27	-0.48
Iraq	3.00	0.09	0.25	-0.01	1.17	1.48	1.08	0.35	-0.50
Israel	1.00	-0.35	-0.18	-0.50	0.28	0.61	0.09	0.17	-0.49
Japan	1.00	-0.22	-0.24	-0.19	0.42	0.46	0.42	0.09	-0.45
Jordan	2.00	-0.17	0.44	-0.39	0.65	2.19	0.36	0.18	-0.49
Kazakhstan	1.00	-0.52	-0.58	-0.47	-0.07	-0.28	0.01	0.19	-0.48
Kuwait	1.00	-0.06	0.23	-0.32	0.50	1.05	0.14	0.21	-0.37
Kyrgyzstan	2.00	-0.33	-0.22	-0.37	0.03	0.24	-0.01	0.10	-0.35
Laos	3.00	-0.16	0.13	-0.21	0.76	0.96	0.79	0.52	-0.52
Lebanon	2.00	-0.35	-0.03	-0.48	0.50	1.49	0.32		-0.57
Malaysia	1.00	0.02	0.24	-0.08	1.05	1.32	1.05	0.24	-0.50
Maldives	2.00	-0.56	-0.26	-0.65	0.32	1.30	0.36	NA	-0.66
Mongolia	2.00	0.07	0.31	-0.08	1.01	1.39	0.89	0.28	-0.47
Myanmar	3.00	-0.09	0.40	-0.20	0.74	1.40	0.64	0.83	-0.48
Nepal	3.00	0.17	0.44	0.02	1.36	1.73	1.15	0.52	-0.50
North Korea	NA	-0.17	-0.16	-0.18	1.91	1.89	1.96	NA	-0.72
Oman	1.00	0.05	0.51	-0.28	1.39	2.66	0.87		-0.56
Pakistan	4.00	0.32	0.35	0.27	1.08	0.96	1.03	0.37	-0.37
Palestine	2.00	-0.18	0.28	-0.31	0.51	1.35	0.40	NA	-0.45
Philippines	2.00	0.19	0.24	0.18	0.58	0.48	0.63	0.19	-0.25
Qatar	1.00	-0.19	0.40	-0.65	0.75	2.65	-0.02	0.15	-0.54
Saudi Arabia	1.00	0.71	1.28	0.39	3.55	5.55	2.99	0.25	-0.62
Singapore	1.00	-0.46	-0.45	-0.46	0.16	0.28	0.16	0.22	-0.54
South Korea	1.00	0.08	0.20	0.04	0.82	0.81	0.93	0.27	-0.40
Sri Lanka	2.00	-0.18	-0.12	-0.22	0.99	0.87	0.99	0.22	-0.59
Syria	3.00	0.88	1.89	0.43	2.68	4.68	2.04	-0.01	-0.49
Tajikistan	3.00	-0.29	-0.03	-0.36	-0.12	0.29	-0.17	0.10	-0.23
Thailand	1.00	0.26	0.68	0.04	1.66	2.26	1.46	0.38	-0.53
Turkey	1.00	-0.28	0.05	-0.46	0.84	1.84	0.55	0.43	-0.61
Turkmenistan	2.00	-0.14	-0.21	-0.06	0.30	0.19	0.41	NA	-0.34
United Arab Emirates	1.00	-0.09	0.14	-0.26	0.62	1.06	0.53	0.31	-0.44
Uzbekistan	2.00	1.17	1.39	1.06	1.93	2.28	1.83	NA	-0.26
Vietnam	2.00	0.82	1.30	0.68	2.96	3.66	3.02	0.48	-0.54
Yemen	4.00	0.50	0.64	0.47	1.46	1.66	1.44	0.19	-0.38

Development, 1. very high (HDI > 0.800, 17 countries), 2. high (0.700 < HDI < 0.799, 16 countries), 3. medium (0.550 < HDI < 0.669, 10 countries), 4. low (HDI < 0.550, 3 countries); HDI, Human Development Index; Incidence, Incidence per 100,000; mortality, Deaths per 100,000; MIR, mortality-to-incidence ratio. Data Source: Global Burden of Disease 2021 study.

**Figure 6:**
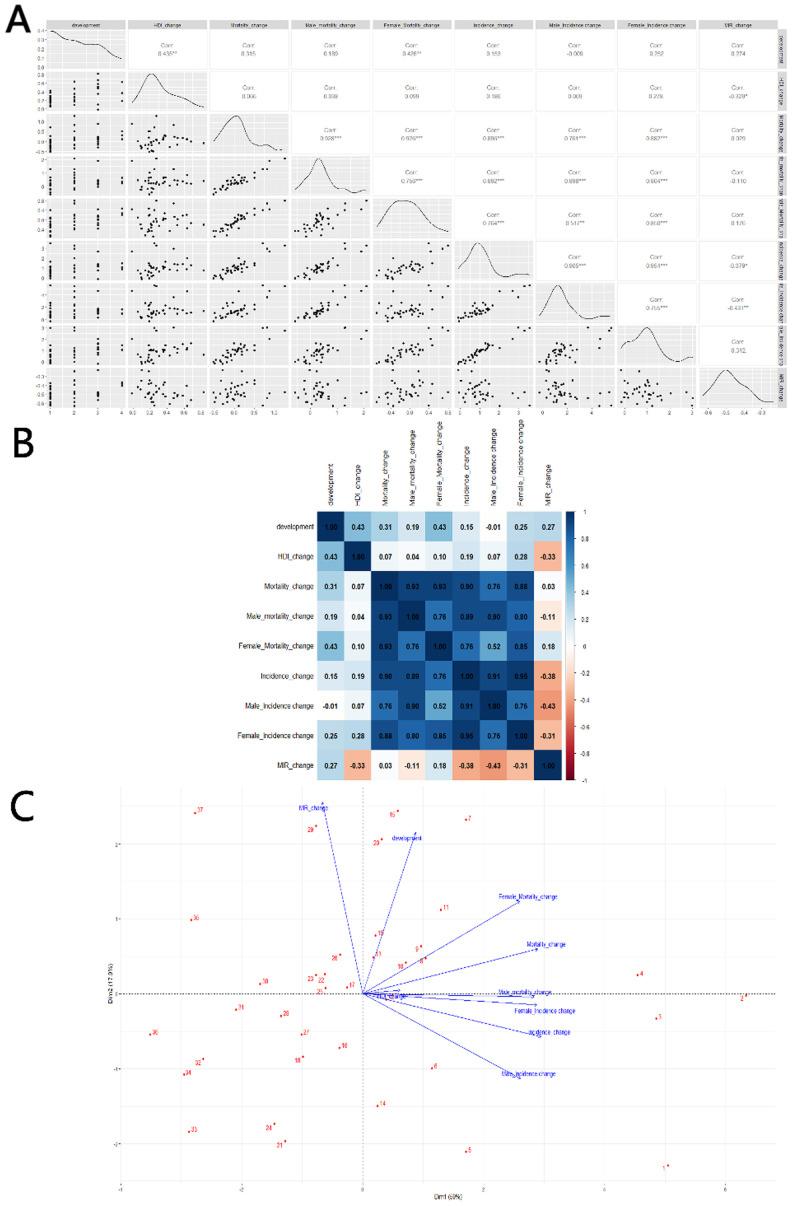
Correlation between HDI change and thyroid cancer Mortality, Incidence and MIR change in 20–54 age: **(A)** scatterplot matrix; **(B)** a correlation heat map; **(C)** principal component analysis plot; HDI, Human Development Index; Incidence, Incidence per 100,000; Mortality, Mortality per 100,000;source; MIR, mortality to incidence ratio; source: Global Burden of Disease 2021 study. An asterisk (*) indicates statistical significance at approximately P = 0.01, two asterisks denote high significance at approximately P = 0.001, and three asterisks signify extreme significance at approximately P = 0.0001.

Changes in HDI are used in the correlation analysis as a reference marker for shifts in the level of social development, as other indicators are not readily available. A scatterplot matrix, correlation heat map, and PCA plot are again used to represent these correlations. In terms of mortality and incidence, changes in HDI and MIR show a significant negative correlation (Corr = -0.329^*^). However, this change is not strongly related to the overall level of social development, whether in very high (HDI > 0.800), high (0.700 < HDI < 0.799), medium (0.550 < HDI < 0.669), or low (HDI < 0.550) HDI countries. The changes in MIR are primarily driven by reductions in morbidity (Corr = -0.379^*^), particularly among men (Corr = -0.431^**^), as shown in **Figure 6**.

## Discussion

This paper examines the burden of TC and its temporal patterns in 47 Asian countries between 1990 and 2021. Using GBD 2021 data, we find that the global burden of TC has increased significantly over the past three decades, with wide inequalities between geographic regions due to varying levels of development. Developed countries (high/very high HDI) have higher incidence rates, while the top 10 countries in terms of mortality are evenly split between developed and less-developed countries. The high prevalence of TC in working-age populations in developed countries can largely be attributed to more frequent screening (11–13).The similarity in TC mortality between developed and developing countries can be explained by several factors. One of the main reasons for higher incidence rates in developed countries is the advancement in diagnostic technology, particularly in more affluent nations. In these countries, the increased detection of small, indolent TCs, which are not typically life-threatening, has led to higher incidence rates without a corresponding rise in mortality. This phenomenon, known as “overdiagnosis,” does not necessarily reflect a higher risk of death from TC. Another reason is that the actual mortality rates for TC remain low and stable across both developed and developing nations. This trend can be attributed to the fact that most thyroid cancers, especially well-differentiated types, have excellent survival rates, regardless of geographical or socioeconomic differences (19).

Overall, the incidence of thyroid cancer in the working-age population and the level of national development have increased in most Asian countries, while about half of the countries have experienced a decline in mortality. The MIR has declined across Asia, suggesting a general improvement in thyroid cancer prognosis, particularly in terms of 5-year survival. However, significant differences in MIR persist between developed and less developed countries. All 10 countries with the lowest MIR are developed, while 8 of the 10 countries with the highest MIR are less developed. Studies suggest that improved healthcare infrastructure in developed countries, including better post-surgical management and the use of radioactive iodine therapy, has contributed to lower mortality rates and better prognoses (19, 20). This indicates that less developed countries need to further enhance their medical services.

In our study, we find that the incidence of TC, particularly in males, is strongly correlated with the level of social development, whereas no such trend is observed in females. This may be attributed to hormonal differences, particularly the effect of estrogen, which may promote tumor growth in women and is not influenced by the social environment (21). Studies have shown that estrogen-related genes are associated with immune regulation, tumor immune evasion, defense systems, signal transduction, the tumor microenvironment, and immune regulation of thyroid cancer. High-risk patients in the immunotherapy dataset had considerably shorter survival times than low-risk patients (22). On the other hand, evidence suggests that men’s participation in regular health checks is increasing, particularly among working-age men. This trend is changing due to greater awareness of the benefits of early detection and preventive care. A study conducted in Guangdong Province, China, which included a predominantly under-45 population, showed that while the detection rate of thyroid nodules was higher in women than in men (13.51% vs. 7.71%), the number of working-age men attending medical check-ups reached 121,883, significantly higher than the 70,680 women, indicating that younger men may now be more willing to undergo medical check-ups (23).

Our study also finds a negative correlation between thyroid cancer mortality and various indices related to social development, with the reduction in mortality primarily coming from women with thyroid cancer. A previous study concluded that overdiagnosis, as a general phenomenon, could improve survival rates in thyroid cancer patients (mortality halved for men and decreased by two-thirds for women) (24). However, this benefit was not evident in some of the more malignant subtypes, which occur more frequently in male patients (25)In both incidence and mortality results, the NMC index shows a statistically significant correlation compared to other indicators. This suggests that NMC has a greater impact on thyroid cancer outcomes, likely because it is an SPI-affiliated indicator reflecting hygiene, health, and nutrition-related parameters. This is particularly relevant as thyroid cancer affects the endocrine system, which is closely related to nutritional factors.

MIR generally declines across Asia but does not show a clear correlation with HDI. However, our findings suggest a noticeable decline in MIR as countries develop, likely due to the scientific and technological advancements described earlier, such as improved management processes and advancing therapies (19, 20). In our study, we find a correlation between changes in MIR and HDI, but statistical significance is lost when HDI is stratified using a four-point scale. This may indicate the limitations of HDI as a sociological parameter, and the challenges of directly applying its stratification method to the medical field. One reason for this could be that HDI is a composite index combining various indicators, and some countries may excel in healthcare but lag in other areas, leading to inflated HDI ratings. Our analytical approach assumes homogeneity in national statistical systems. In regions with limited healthcare administration capacity, longitudinal patient tracking becomes operationally challenging, introducing potential bias in outcome reporting. The implications for data validity are examined in Section Limitation. In our analysis of factors associated with changes in thyroid cancer incidence and mortality, we primarily use HDI as the independent variable. This is partly because other indicators are not readily available and partly because previous literature has already established an association between cancer burden and HDI, confirming the reliability of such comparisons (9).

Notably, despite the marked rise in TC incidence, we have observed a concomitant increase in working-age mortality. This phenomenon may reflect both improved attribution of previously misclassified deaths and potential negative shifts in either healthcare system capacity or disease subtype distribution over time—though further empirical validation is warranted.

DOD is associated with a high incidence of TC but does not show a clear correlation with mortality. Previous studies have suggested a potential link between physician density and patient mortality in highly malignant tumors, such as bladder and prostate cancer, but without clear statistical significance (26). We believe that DOD primarily reflects primary care equity, as a higher density of physicians facilitates the screening of early-stage cancers (27). However, for malignant TC subtypes, which are more lethal, prognosis likely depends more on the quality of treatment and care at advanced medical centers.

### Limitation

First, estimates of the burden of TC are derived from the GBD 2021 study, and the quality of these estimates heavily depends on the availability of cancer registry data. In many low-income countries, cancer registry coverage is incomplete, leading to gaps in data. As a result, spatial and temporal data from neighboring regions are used to estimate the global burden of disease. This lack of cancer registry data creates large uncertainty intervals, which may be too broad to provide meaningful policy implications for these countries. Second, in less developed countries, the estimates presented here may be biased downward due to underreporting or misclassification of cancer deaths. Third, it is well known that cancers detected early (clinical stage 1) are less severe and less life-threatening than those detected at later stages (clinical stage 3 or 4); however, no estimates are provided for cases diagnosed at different clinical stages. Fourth, in some Middle Eastern and Southeast Asian countries, prolonged wars and conflicts have weakened grassroots health management and organizations, leading to the inclusion of data only from larger cities, which introduces bias. Additionally, in many countries, weak governance has resulted in a lack of effective health infrastructure, with deaths due to war, plague, or starvation potentially reducing the reported number of cancer deaths. As the GBD database aggregates fragmentary institutional records, its national-level estimates risk misattributing institutional-level patterns to entire populations. Lastly, some countries are missing key data. For example, the DPRK is unable to provide valid parameters such as the HDI, and others, like Bhutan, lack HDI data for 1990, which affects rate-of-change calculations. These missing data points can introduce bias into the results.

## Conclusions

The increasing burden of TC in the working-age population across Asia has led to a rise in incidence, particularly in developed countries. However, overall prognosis has gradually improved, likely due to advancements in human development that enable earlier detection, greater awareness, and improved treatment modalities. Based on past trends, TC incidence is expected to increase further, driven by irregular work schedules, unhealthy lifestyles, poor diets, hormone disruption, and exposure to ionizing radiation within the working-age population. In less developed countries, the lack of early detection, late diagnosis, and the absence of active surveillance systems are expected to place significant strain on healthcare systems with limited resources. In these countries, TC exhibits a higher mortality rate, which can be attributed to late diagnosis, misdiagnosis, limited access to skilled surgeons and effective treatments, as well as the high cost of care and lack of health insurance coverage. These challenges contribute to the greater burden faced by cancer patients in these regions. Given the pronounced gender differences in thyroid cancer, increased awareness of the need for ultrasound screenings in working-age men is crucial. Regardless of gender, a comprehensive approach—ranging from regular check-ups and prevention to individualized treatment—will help improve prognosis. Due to the significant developmental disparities between Asian countries, there is an urgent need to enhance healthcare capacity and education in underdeveloped regions. Additionally, the hidden costs of taxes and logistics for imported medical products further increase the burden on patients. To address these challenges, less developed countries should prioritize investment in international cooperation and local research and development, focusing on strengthening primary healthcare systems and developing advanced healthcare centers.

## Data Availability

The datasets presented in this study can be found in online repositories. The names of the repository/repositories and accession number(s) can be found in the article/**Supplementary Material**.
